# Transmembrane TNF and Its Receptors TNFR1 and TNFR2 in Mycobacterial Infections

**DOI:** 10.3390/ijms22115461

**Published:** 2021-05-22

**Authors:** Andy Ruiz, Yadira Palacios, Irene Garcia, Leslie Chavez-Galan

**Affiliations:** 1 Laboratory of Integrative Immunology, Instituto Nacional de Enfermedades Respiratorias “Ismael Cosio Villegas”, Mexico City 14080, Mexico; andy.ruiz@iner.gob.mx (A.R.); yadpal@gmail.com (Y.P.); 2 Department of Pathology and Immunology, Centre Medical Universitaire (CMU), Faculty of Medicine, University of Geneva, 1211 Geneva, Switzerland; Irene.Garcia-Gabay@unige.ch

**Keywords:** tumor necrosis factor, TNF receptors, mycobacterial infections, tumor necrosis factor-α converting enzyme (TACE)

## Abstract

Tumor necrosis factor (TNF) is one of the main cytokines regulating a pro-inflammatory environment. It has been related to several cell functions, for instance, phagocytosis, apoptosis, proliferation, mitochondrial dynamic. Moreover, during mycobacterial infections, TNF plays an essential role to maintain granuloma formation. Several effector mechanisms have been implicated according to the interactions of the two active forms, soluble TNF (solTNF) and transmembrane TNF (tmTNF), with their receptors TNFR1 and TNFR2. We review the impact of these interactions in the context of mycobacterial infections. TNF is tightly regulated by binding to receptors, however, during mycobacterial infections, upstream activation signalling pathways may be influenced by key regulatory factors either at the membrane or cytosol level. Detailing the structure and activation pathways used by TNF and its receptors, such as its interaction with solTNF/TNFRs versus tmTNF/TNFRs, may bring a better understanding of the molecular mechanisms involved in activation pathways which can be helpful for the development of new therapies aimed at being more efficient against mycobacterial infections.

## 1. Tumor Necrosis Factor (TNF) and Tumor Necrosis Factor-α Converting Enzyme (TACE)

TNF was described for the first time in the middle of 1970; it is a polypeptide considered a potent pro-inflammatory cytokine and encoded in the major histocompatibility complex in human and mice, and it is produced by immune system cells both myeloid and lymphoid origin [1,2,3].

TNF is synthesized as a monomeric protein and stored in vesicles, which use the route of rough endoplasmic reticulum (ER) to cross the cytoplasm. TNF monomers form a compact trimer through non-covalent interactions; the TNF-trimeric has high thermodynamic stability, the molecular mass of the human TNF is 50.4-kDa and murine TNF 50-kDa [4,5]. Active TNF is also expressed as a trimeric transmembrane form on the cell surface (hereafter tmTNF); after an activation stimulus, tmTNF is proteolytically processed, and a soluble form is released (hereafter solTNF); both tmTNF and solTNF display physiological functions [6].

TACE or also called A Disintegrin and Metalloproteinase (ADAM) domain 17 (ADAM17), cleaves tmTNF between residues Ala76 and Val77 to obtain solTNF [7]. TACE is the only, or at least the major sheddase of TNF in vivo, other ADAM family members such as ADAM10, ADAM9, and ADAM19 have been shown to shed TNF only in vitro and the cleavage site does not match with the physiologically relevant site [8]. TACE is not a TNF specific protease; several other proteins such as transforming growth factor-beta (TGF-β), beta-amyloid precursor protein, and TNF receptors are released by the TACE action [8,9]. It has been well documented that TACE also mediates the cleavage of the receptor Angiotensin-converting enzyme-2, a receptor used by viruses such as SARS-CoV and SARS-CoV2 to infect the cells [10,11,12].

It is currently well established that TACE is necessary to deliver solTNF; however, how its catalytic activity is activated or regulated is still limited. Probably, the presence of a functional TACE is dependent on the action of multiple molecules. For instance, in TGF-β-mediated TACE activation, when TGF-β binds to its receptors, the sarcoma kinase (Src) molecule is phosphorylated, mediating NOX 1 activation and producing reactive oxygen species (ROS), which is finally activating TACE [13]. The TNF-dependent pathway activates the nuclear factor kappa-light-chain-enhancer of activated B cells (NF-κB) signalling, whereas the Uev1A-Ubc13 complex catalyzes the ubiquitination of RHBDF2, which is a crucial factor to promote TACE maturation. Consequently, Uev1A-Ubc13 inhibition interferes with RHBDF2-promoted TACE maturation [14].

Three mechanisms proposed to explain how the TACE’ catalytic activity is regulated are discussed below.

**Figure 1:**
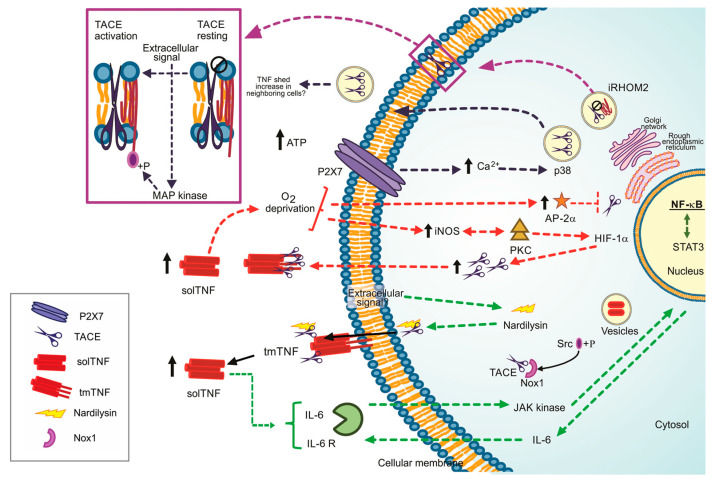
Regulation pathways to the TNF/TACE axis, ways to lead the TACE activation. Dotted green line: Nardilysin-dependent pathways. Dotted red line: iNOS/AP-2a-dependent pathways. +P Phosphorylate form. Dotted blue black line: P2X7 dependent pathway, Dotted purple line: iRHOM2 as the TACE maturation promoter. Black arrow up: increased level.

(1) Nardilysin (NRD)-dependent pathway: NRD is a metalloendopeptidase of the M16 family. NRD binds to TACE (at cytosol or extracellular level) and potentiates its catalytic activity, a process promoted by phorbol esters. The NRD gene’s overexpression increases TNF and induces ADAM10 activation, suggesting that the high TNF shedding could be due to the function of both TACE and ADAM10 [15,16] (Figure 1, dotted green line). Although the TNF shedding by ADAM10 has been observed only as an in-vitro process, using cultures of TACE-deficient fibroblasts, solTNF was detected, suggesting that ADAM10 is the TNF shedder when TACE is not present [17].

TNF can promote cancer cells’ proliferation [18]. NRD increases TNF shedding; consequently, NF-κB signalling pathway and pro-inflammatory microenvironment, characterized by the presence of interleukin (IL)-1β, IL-6 and prostaglandin E2 are activated. In turn, STAT3 is phosphorylated by the autocrine function of IL-6, and growth-related genes are upregulated [19] (Figure 1, dotted green line). Several questions remain open, for instance, if a specific extracellular signal is necessary or not to induce the complex NRD/TACE or if ADAM10 can or not cleave TNF in vivo.

(2) Oxygen deprivation-dependent pathway. Reports suggested that oxygen deprivation induces increased TACE expression and, consequently, high solTNF levels [20]. By this pathway, the protein kinase C (PKC) and inducible nitric oxide synthase (iNOS) induce a nuclear accumulation of NF-κB and hypoxic-inducible factor-1 subunit α (HIF-1α) to stimulate TACE promotor activity and solTNF increase by an autocrine function, thus the shedding of TNF could be perpetuated [21,22] (Figure 1, dotted red line).

Reports also suggested that solTNF upregulates the transcription factor AP-2α. However, the TACE promoter contains an AP-2α binding sequence, and it can bind in a TNF-dependent manner, indicating that solTNF downregulates TACE expression and function because AP-2α is enhanced [23] (Figure 1, dotted red line). The role played by AP-2α in the synthesis and function of TACE is controversial. Evidence showed that TNF induces caspase-6 activation, which in turn cleaves AP-2α, suggesting that TNF downregulates AP-2α by a caspase 6-dependent pathway [24]. It is possible that the concentration of solTNF and tmTNF, or maybe one of the receptors, could be responsible for determining the up-or down-regulation of AP-2α and its consequent role.

(3) P2 and iRHOM2 pathways. The P2 purinergic receptors have bi-functional effects on TNF release. On the one hand, P2X receptor activation attenuates TNF release and simultaneously, on the other hand, P2Y induces TNF release [25]. ATP induces intracellular Ca^2+^ rise by P2X7-dependent pathway, activating kinase p38 and finally promoting TACE’s release into exosomes. It could be a mechanism to shed membrane proteins to neighboring cells, thus propagating inflammation [26] (Figure 1, dotted black line).

iRHOM2 (rhomboid 5 homolog 2) or RHBDF2 is a member of the rhomboid protein family found in the ER. iRHOM2 is considered an essential regulator for the crosstalk TNF/TACE as it helps TACE to get the plasma membrane [27]. Data indicate that Ubiquitin (Ub)-conjugating enzyme variant 1A (Uev1A) polyubiquitinates iRHOM2 promoting TACE maturation (Figure 1, dotted purple line). However, the role of iRHOM2 is not limited to induce TACE maturation. The phosphorylation of the iRHOM2 cytoplasmic tail by MAP kinases (p38, JNK and ERK1/2) is a crucial step to expose TACE proteolytic site activating its sheddase function [14,28] (Figure 1, purple box).

## 2. Tumor Necrosis Factor Receptors

Two receptors have been identified to mediate interactions with TNF, tumor necrosis factor receptor 1 (TNFR1), also called CD120a and p55 (its molecular weight is 55 kDa), and tumor necrosis factor receptor 2 (TNFR2), also called CD120b and p75 (its molecular weight is 75 kDa) [29]. TNFR1 and TNFR2 are not specific to TNF, they interact also with lymphotoxin alpha (LTα, previously known as TNFβ). LTα is a cytokine closely related to TNF, activated by similar stimuli than those activating TNF, produced mainly by lymphoid cells in a soluble form and can combine with LTβ interacting with another different receptor, LTβR [30].

TNFR1 and TNFR2 are on the cellular membrane or in a soluble form following TACE activation; their cytoplasmic domains are unrelated, and intracellular signalling pathways are independent. TNFR1 is involved in cytotoxicity, whereas TNFR2 plays a role in cytotoxicity and proliferation [31,32]. As described in the previous section, the exact mechanism involving TACE in the shedding of TNFR1 and TNFR2 is still unclear.

### 2.1. Tumor Necrosis Factor Receptor 1

**Figure 2:**
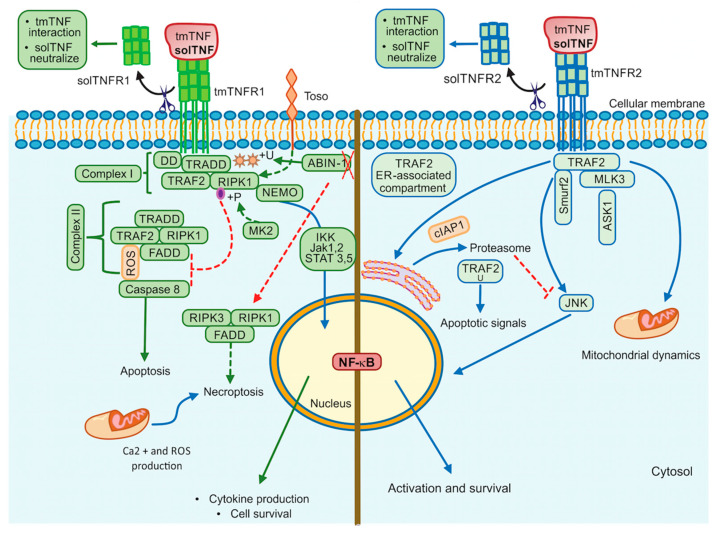
TNFR1 and TNFR2 activation pathways. TNFR1 signalling is involved in cell death events and inflammatory processes by classical NF-kB, whereas TNRF2 triggers signalling that may activate classical or non-classical NF-kB pathway. Additionally, TNFR2 impacts mitochondrial dynamics. ABIN-1: Ubiquitin binding protein, DD: Death domain, FADD: Fas-associated death domain, IKK: IkappaB kinase, Jak: Janus kinase protein, NEMO: The NF-κB essential modulator (or IKKγ), RIPK1: Receptor interacting protein kinase 1, RIPK3: Receptor interacting protein kinase 3, ROS: Reactive oxygen species, STAT: Signal transducer and activator of transcription proteins, Toso: Human Fcμ receptor (hFCMR) or FAIM3, TRADD: TNFR1-associated death domain, TRAF2: TNFR-associated factor 2, +P: Phosphorylation, +U: Ubiquitination. Continuous lines green and blue: activation signalling. Dotted lines: inhibition signalling. A red cross: absence.

TNFR1 contains a cytoplasmic region designed death domain (DD), which initiates a signal of cytotoxicity with homology to the intracellular domain of Fas antigen [33]. TNFR1 activates different signalling pathways, including neutrophil migration, complement pathway, regulation of other cytokines and chemokines, adhesion molecules and their receptors, generally promoting inflammatory responses [34]. Several molecules were described as essential for NF-κB activation through a TNFR1-dependent pathway, for example, TRADD (TNFR1-associated death domain), RIPK1 (receptor-interacting protein kinase 1, also known as RIP1), TRAF2 (TNFR-associated factor 2) and FADD (Fas-associated death domain) [35]. The TNF/tmTNFR1 complex induces activation signalling pathways leading to opposite effects such as cell survival and cell death. Once TNF binds tmTNFR1, TRADD, RIPK1, TRAF2 and TAK1 molecules are recruited near DD and complex I. This complex I mediates the activation of MAPK and NF-κB, promoting cell survival. To activate this pathway, the phosphorylation of Jak-(Janus kinase)1 and Jak2, STAT-(signal transducer and activator of transcription)3 and STAT5 are required [36,37]. RIPK1 (RIPK1^U^) is ubiquitinated to recruit Ikappa B kinase (IKK), and then there is a binding between RIPK1^U^/NEMO (regulatory subunit IKKγ) to finally activate NF-κB [38] (Figure 2, left). However, if TRADD, RIPK1 and TRAF2 are dissociated from DD, they can interact with FADD, assembling the complex II that activates Caspase-8, inducing cell death. It has been suggested that to induce a full caspase 8-activation, ROS generation is required upstream and downstream of complex II [37,39] (Figure 2, left).

The interactions of solTNF or tmTNF with tmTNFR1 are also crucial to trigger apoptotic signals. It has been recently shown that tmTNF induces the binding of STAT1 to a region spanning amino acids 319–337 of tmTNFR1. STAT1-phosphorylation (at the serine residue in position 727) favors its binding to TRADD and FADD, promoting apoptosis but not NF-κB activation [40]. Thus, the regulatory balance between survival versus death by the TNFR1 pathway is crucial to maintain homeostasis, and several authors suggested that TNFR1-mediated-signal transduction includes a checkpoint resulting in cell death when the signal activating NF-κB fails. Although it is not yet clarified how this checkpoint is controlled, experimental evidence suggested that RIPK1 is a crucial target to regulate this balance. It was reported that IKKα/IKKβ mediates direct phosphorylation of RIPK1 as the last step regulating cell death [41].

Toso, also known as FAIM3 (Fas apoptosis inhibitory molecule 3) or FcμR, is a transmembrane protein with a negative regulatory function, which promotes the ubiquitination of RIP1 inducing NF-κB activation and consequently cell survival [42], affecting caspase 8 activation [43]. Another molecule that has been involved in cell death is MAPKAP kinase-2 (MK2) [44]. If the effector MK2 induces phosphorylation of the kinase RIPK1, the binding with FADD/Caspase 8 is inhibited, thus complex-II-dependent cell death is blocked [44,45]. TNFR1-mediated cell survival may depend on ubiquitination and phosphorylation of RIPK1 (Figure 2, left).

Additionally, TNF/tmTNFR1 pathway can induce cell death called necroptosis as the assembly of FADD, RIPK1, and RIPK3 form the complex called necrosome [46]. Roca and collaborators [47], have shown in zebrafish or in human macrophages (infected with *Mycobacterium marinum* or *Mycobacterium tuberculosis*) that an excess of TNF triggers necrosis through TNF-RIPK1-RIPK3 interactions increasing the production of ROS, cyclophilin D (mitochondrial matrix protein), BID and BAX (pro-apoptotic proteins) [48]. The same group has recently reported that TNF triggers the production of ROS activating cyclophilin D (mitochondrial matrix protein) and leading to BID and BAX (pro-apoptotic proteins) activation, which results in mitochondrial Ca^2+^ overload through ER ryanodine receptor and necrosis [47].

The Ubiquitin-binding protein ABIN-1 is critical for the activation of RIPK1 [49]. This protein is recruited into the complex I and plays a critical role in the control of ubiquitylation and deubiquitylation. It has been proposed that ABIN-1 deficiency reduces the recruitment of A20 molecule (negative regulator of NFκB), and consequently, there are ubiquitylation and activation of RIPK1 to mediate necroptosis [49] (Figure 2, left).

### 2.2. Tumor Necrosis Factor Receptor 2

Although the tmTNFR2 signalling pathway has been mainly implicated in cell proliferation, activation and survival, data have also reported to transduce apoptotic signal under specific models and potentiate TNFR1-induced cell death [50,51].

Two molecules with the ability to interact with the TNFR2 cytoplasmic domain and to induce signalling were reported and called TNF receptor-associated factor 1 (TRAF1) and TRAF2 [52]. TNFR2 signalling was first simplified as a pathway where TRAF2 induces c-Jun-N-terminal kinase (JNK) activation, using the apoptosis signal-regulating kinase 1 (ASK1) as a mediator to activate NF-κB and facilitating anti-apoptotic signals [53].

JNK plays a fundamental role to determine the outcome of the TNFR2 pathway. It has been shown that after TNF/tmTNFR2 engagement, TRAF2 and the inhibitor of apoptosis molecules called cIAP1 (molecule to mediate the TRAF2 ubiquitination) are recruited to the TNFR2 cytoplasmic domain, and later it is translocated to an ER-associated compartment, where TRAF2 ubiquitination occurs [54,55] (Figure 2, right). The ER is sensitive to homeostasis alterations favoring the accumulation of misfolded proteins, which trigger ER stress and promote apoptotic cell death [56]. Thus, after ER-stress, TRAF2 forms a complex with the ER-stress sensor called IRE1 and interacts with procaspase-12 promoting its activation [57]. It has also been proposed that ER-stress induces expression of TNF in an IRE1- and NF-κB-dependent manner, and TRAF2 is decreased; these together inhibit the JNK activation and makes cells susceptible to cell death [58]. Those data support evidence for a relevant role of the ER in an additional apoptotic control point of the TNFR2 pathway.

It has also been proposed that following tmTNFR2 activation, the E3 ubiquitin ligase Smurf2 forms a ternary complex with tmTNFR2 and TRAF2, inducing relocalization of TNFR2 to the insoluble membrane/cytoskeletal fraction promoting the JNK activation [59]. Mixed lineage kinase 3 (MLK3), a mitogen-activated protein kinase kinase kinase (MAP3K) required for optimal activation of JNK signalling, has been shown to associate with TRAF2, TRAF5 and TRAF6. However, only TRAF2 induces the kinase activity of MLK3 by conjugating with polyubiquitin chains to activate JNK [60,61].

Finally, there is controversial evidence about the role of TNF on mitochondrial integrity. Reports have shown that TNF causes mitochondrial fragmentation (fission), but other authors suggested that TNF stimulates mitochondrial biogenesis [62,63]. In human airway smooth muscle (hASM) non-asthmatic cells, fission was associated with an increased level of Drp1 (Dynamin related protein (1) and the decreased level of Mfn2 (GTPase Mitofusin (2), and TNF was involved in Mfn2 reduction [64]. Recently, it was reported that hASM cells exposed to TNF showed fission and mitochondrial biogenesis, with increased organelle volume density, cell proliferation, reduction of Ca^2+^ influx, and decrease of O2 consumption per mitochondrion [65]. In this regard, the TNFR2-activation mediates interactions between Stat3 (signal transducer and activator of transcription (3) and Re1A (Stat-Re1A protein) as the Stat3/Re1A complex interacts with two lysine residues within Stat3 that is acetylated by p300 and OPA1 (optic atrophy 1) expression is increased, suggesting an enhanced mitochondrial fusion [66].

In summary, the tmTNFR2 pathway is tightly regulated depending on different cellular organelles, which are alternative for specific cellular systems when TNF is produced in inappropriate concentration. These mechanisms can help to develop novel therapeutic targets for treating or preventing diseases where TNF expression is dysregulated.

## 3. tmTNF Signalling Pathway

As previously discussed, tmTNF is a stable homotrimer that is cleaved by TACE and released as solTNF. The first publications on tmTNF suggested that tmTNF interacts primarily with TNFR2, whereas solTNF binds mainly to TNFR1 [67,68]. However, we need to consider that the tmTNF form that showed the specific interaction with TNFR2 are artificial because that tmTNF form contains mutations, which are not found in the native tmTNF molecule, and it is identical to solTNF. Also, tmTNF mutations to be retained on the cell membrane can differ, resulting in different outcome after an infection in vivo and in vitro [30,69]. At present, no data have been reported, excluding the interaction of TNFR1 with tmTNF. Many data have reported on TNFR1-tmTNF interactions in vivo and in vitro and, in particular, in the context of mycobacterial infections using tmTNF mutant mice that will be treated in this review.

**Figure 3:**
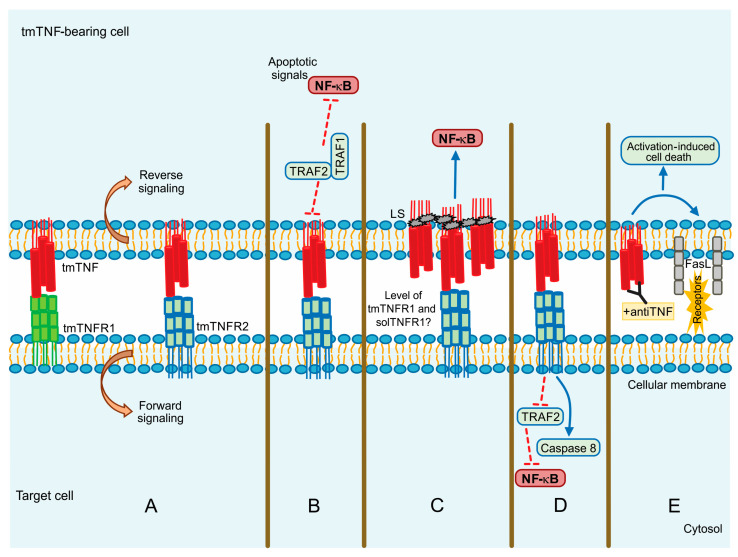
tmTNF signalling pathway. (**A**) Forward and reverse signalling; (**B**) Apoptotic signal by TRAF1 and TRAF2; (**C**) Direct NF-kB activation by LS (leader sequence) in tmTNF; (**D**) Actin involvement in tm-TNF transduction; (**E**) Activation Induced Cell Death (AICD) by upregulation of FASL (CD95L). Continuous blue lines: activation signalling. Dotted red lines: inhibition signalling.

tmTNF is a molecule that induces crosstalk between tmTNF-bearing cells and tmTNFR-bearing cells signalling both as a ligand and as a receptor. This means that tmTNF not only mediates the forward signal to the target cell but also mediates the reverse signalling inside the tmTNF-bearing cell [69] (Figure 3A).

Evidence suggests that although solTNF and tmTNF mediate cytotoxicity, tmTNF can exert apoptosis on solTNF-resistant cells. Using a model of TNF-resistant cells (the HL-60 cell line), it has been reported that tmTNF promotes the interaction with TRAF1 and TRAF2. TRAF1 plays a suppressor role in blocking the translocation of TRAF2 from the cytoplasm to the cell membrane. Consequently, NF-κB activation is inhibited resulting in cell death (Figure 3B) [70]. The same group has also proposed that forward signalling can result in opposite activities since tmTNF acting as a receptor promotes NF-κB activation, whereas acting as a ligand inhibits NF-κB activity [71].

It has been reported that MCF-7 human tumor cells with a high expression level of tmTNF are resistant to cell death associated with solTNF and constitutive NF-κB activation. tmTNF contains a leader sequence (LS) in the cytoplasmic segment (76 amino acid residues) through which tmTNF is anchored into the membrane. LS appears to affect both forward and reverse signalling, and it seems that LS induces directly the constitutive activation of NF-κB [72,73] (Figure 3C).

Using a pleural cell model, we have previously shown that tmTNF but not solTNF controls the expression of TNFR2 on myeloid cells expressing tmTNF, TNFR1 also contributes but in a minor way. Besides, the inflammatory process of BCG-induced pleurisy was downregulated mainly by tmTNF and TNFR2 [74]. Recent studies have shown that tmTNF efficiently activates both TNFR1 and TNFR2, but solTNF interacts and activates TNFR1 [75,76]. In several liver injury models, it has been shown that only solTNF causes liver toxicity but not tmTNF which can protect the host against mycobacterial infections [77,78]. Recently, we reported that TNFR1 is necessary to recruit myeloid cells, while TNFR2 is implicated in cell activation after BCG-induced pleural infection [78,79].

The actin cytoskeleton plays an essential role in different functions of the cell, for instance, intracellular trafficking and cellular contractility, but also it has been recognized that the actin’s dynamic structure is involved in apoptosis and necrosis [80]. The actin’s dynamic is another molecular mechanism by which the signalling activated can differ between both forms of TNF. Data have shown that solTNF induces actin depolymerization and morphological changes through ERK activation and p38 MAPK inducing cell death [81]. In contrast, tmTNF does not affect the state of actin microfilaments; apparently, actin is involved in tmTNF-mediated signal transduction by uncoupling TRAF2 and cFLIP from TNFR2 and consequently activating caspase-8 to induce apoptosis and inhibiting NF-κB activation (Figure 3D) [82].

Activation-induced cell death (AICD) plays a role in regulating peripheral immune tolerance by deleting overactivated or autoreactive T cells. It has been described that NF-κB is required to mediate the expression of the pro-apoptotic molecule called Fas ligand (FasL or CD95L) inducing AICD [83]. It was recently reported that when tmTNF functions as a receptor, using an anti-TNF polyclonal antibody to trigger reverse signalling, tmTNF can upregulate FasL expression and, consequently, increase AICD. Moreover, tmTNF-dependent reverse signalling also significantly increases several ligands, including TNFRs and Fas (Figure 3E) [84].

## 4. TNF/TNFR1/TNFR2 Inhibitors

Diverse proposals of anti-TNF therapies targeting the cytokine or the receptors are underway [85,86]. Although the approved TNF therapies present several side effects, these are efficient treatment options for controlling inflammatory diseases [87]. Both tmTNF and solTNF are biologically active, and the balance between the two forms is influenced by the cell type and its activation state. The long-term use of various TNF pathway inhibitors, like monoclonal antibodies, to treat diseases such as rheumatoid arthritis or Crohn’s disease can modify the regulatory activity of T cells and lead to an increased susceptibility to bacterial infection. Mycobacterial infections are mainly due to *Mycobacterium tuberculosis* (*M. tuberculosis*) and *Mycobacterium bovis* (*M. bovis*), and also, there are unwanted effects such as sepsis autoimmunity and neurodegeneration.

Among the most used inhibitors are Etanercept, a TNFR2 dimeric fusion protein, followed by monoclonal antibodies Adalimumab and Infliximab. Their therapeutic efficacy is dependent on the interaction of the Fc region of the anti-TNF IgG and the FcγR expressed on the cell surface. Moreover, these antibodies have been related to a specific risk of extrapulmonary and disseminated infections [88].

New therapeutic strategies are proposed to target the TNF axis with a minimum risk of *M. tuberculosis* reactivation. For instance, the new design of a hypo-fucosylated form of Adalimumab, which was found to have better healing properties due to high affinity for FcγRIII and induction of CD206+ macrophages without apparent adverse effects. However, validation in large cohorts of patients to verify the clinical response is required [89]. Sultana and Bishayi have used to neutralize or selectively inhibit TNFR1 or TNFR2 in a model of *Staphylococcus aureus*-induced septic arthritis; authors showed that the levels of pro and anti-inflammatory cytokines were modulated via the NF-kB and JNK signalling, which favored an increase of iNOS and RANKL and reduced recruitment of phagocytes at the site of inflammation, and subsequently decreased the generation of ROS and septic arthritis [90].

Additional studies suggest that the combination of conformational analysis and molecular coupling studies for a cyclic peptide inhibitor of human TNF and TNFR1 could result in a rational design of a new moderate inhibitor of TNF-TNFR1 interaction. The combination of these cyclic peptides was shown to improve severity in rat colitis models, depending on the use of TNF-binding cyclic peptide (TBCP) or TNFR1-binding (TRBCP) [91]. Molecules such as progranulin (PGRN), a secretory growth factor that binds directly to TNFR1 and TNFR2, an endogenous TNF antagonist, induces T regulatory cells increasing IL-10 production, activates ERK2 pathway and suppresses the stimulation of IL-1β and TLR4 by binding to TNFR1 [92].

Dominant-negative TNF molecules selectively blocking solTNF but not tmTNF molecules were designed and reported to prevent inflammatory process mainly mediated by solTNF [93]. These selective inhibitors of solTNF were shown to protect mice from acute liver injury while preserving the activity of tmTNF required for host defence mechanisms against mycobacterial infections. In contrast, non-selective inhibitors of both solTNF and tmTNF, such as Etanercept, suppressed immunity to mycobacterial infections [94,95].

Spohn et al. [96] reported virus-like particle-based vaccine selectively targeting solTNF by generating anti-TNF antibodies which protected mice from arthritis without affecting reactivation of latent tuberculosis [96]. In this regard, Zhang et al. [97] designed a TNF epitope scaffold immunogen, the DTNF7 vaccine using the diphtheria toxin transmembrane domain (referred to as DTT as scaffold). The grafted TNF epitope is wholly exposed to the surface and presents in a native conformation, while the rigid helical structure of DTT is minimally disturbed, which induces a sustained antibody response since the immunogen is highly stable. This TNF epitope-scaffold immunogen induced sustained antibody responses in a mouse model of collagen-induced arthritis [98]. These authors proposed a selective modulation of TNFR1 and TNFR2 may represent advantages over TNF general inhibition because it allows the preservation of the non-target receptor’s function and reduces the side effects [97]. These results could represent a potential application to reduce the risk of latent tuberculosis (TB) activation.

A summary of TNFR1 and TNFR2 modulators is presented in Table 1. Other efforts have focused on designing and developing small selective inhibitors of the TNF converting enzyme (TACE). Zinc Binding Groups (ZBG) have been considered to play an essential role in determining the efficacy, selectivity and TACE Inhibitor Toxicity. Some of the TACE inhibitors already developed have been used in topical treatments for dermatological conditions such as psoriasis and acne, limiting their systemic toxicity. Research is currently underway to design monoclonal antibody inhibitors from TACE [99].

The use of new biological is currently limited, and it is due mainly to the high cost of clinical studies and potential risk of side effects in human. Animal models for preclinical studies have been developed, for example mice expressing human TNF molecules to overcome the limitation of analyzing a human TNF inhibitor in mouse [100]. However, efforts must be conducted to elucidate the reliability of novel drugs, models of study, characterization of signalling pathways in the context of mycobacterial infection the side effects as reactivation of latent infection.

## 5. Contribution of tmTNF in the Control of Mycobacterial Infections

Tuberculosis (TB) is an infectious disease, which is the leading cause of death worldwide from a single infectious agent; the World Health Organization has estimated that in 2019 worldwide, 10 million people fell ill with TB (range, 8.9–11.0 million) and 1.2 million deaths [115]. The causative agent of TB is the bacillus *M. tuberculosis*; the current vaccine used against TB is the Bacillus Calmette-Guérin (BCG), which is derived from another mycobacterium called *M. bovis*. BCG vaccine provides adequate protection against childhood TB, but the level of protection against adult pulmonary TB can be variable [116].

TNF plays a critical role in host defense mechanisms against mycobacterial infections as shown by many research groups using different models of TNF and TNFRs deficient mice [30]. In vitro, infected macrophages produce TNF, and the amount is related to bacterial virulence and infection doses. Even with very low doses of BCG, macrophages can produce TNF [117]. Experimental data have shown that the absence of TNF or TNF receptors correlates with an exacerbated inflammatory process and an impaired bacterial clearance that culminates with disseminated mycobacterial infection and host’ death [75].

It has been recently reported that *M. tuberculosis* DNA (MtbDNA) is recognized by murine macrophages, which leads to autophagy induction, TLR-9 expression, and considerable TNF production. Interestingly, only M1 macrophages were fully responsive to MtbDNA [118]. Several reports suggest a modulation in macrophages during *M. tuberculosis* infection where TNF, TLRs and autophagy are involved [119,120].

Most TNF activities have been attributed to solTNF form. At present, the contribution of tmTNF in protective immunity against mycobacterial infections has been also well analyzed by using mutant mice that only express tmTNF and not solTNF. However, as previously discussed, these tmTNF are modified molecules that are retained at the cell membrane representing a model system but not the native tmTNF molecule.

**Figure 4:**
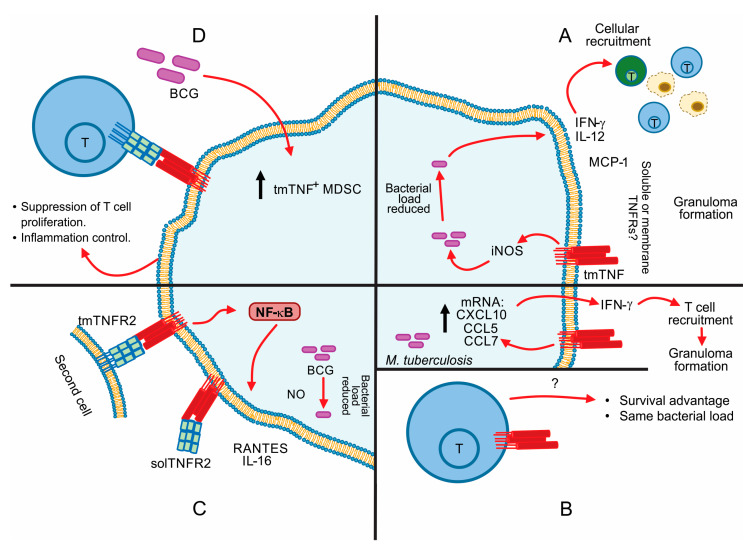
tmTNF in mycobacterial infection. (**A**) tmTNF and iNOS in granulomas promote the secretion of Th1 chemokines and cytokines; (**B**) Up: *M. tuberculosis* acute-phase infection in mice. Below: tmTNF expression in T cells during *M. tuberculosis* infection. (**C**) tmTNF regulation by tmTNFR2 or solTNFR2; (**D**) tmTNF expression on MDSC (Myeloid-Derived Suppressor Cells) leads to specific interaction with tmTNFR2. Black arrow up: increased level.

In 2002, Olleros et al. [121] reported that tmTNF might act as a receptor upon binding of soluble or membrane TNFRs to develop efficient bactericidal mechanisms. Transgenic mice expressing only tmTNF but not solTNF and LTα were able to activate an efficient immune response against BCG and acute *M. tuberculosis.* tmTNF was sufficient to sustain the cellular activation and to reduce bacterial BCG load by inducing the granuloma formation, and IFN-γ expression although the amounts were lower than those induced when solTNF was expressed [121]. Using these mutant mice, tmTNF expression was also associated with an efficient granuloma formation with activation of iNOS as well as the induction of local and systemic Th1-type cytokines such as IFN-γ and chemokines such as MCP-1 (Figure 4A). However, these mice expressing tmTNF but not solTNF and LTα were able to survive to BCG and acute *M. tuberculosis* infection but not to chronic *M. tuberculosis* infection as these mice developed an exacerbated inflammation [122].

In a different mouse model of tmTNF knocking (KI) mice, Saunders et al. [123] showed that mice expressing stable tmTNF (TNF-membrane bound without TACE cleavage site), but not solTNF, were able to contain bacterial growth for over 16 weeks and developed antigen-specific T cell response with compact granulomas as wild-type (WT) mice. This work reported that during the acute-phase infection (first infection 12 weeks), tmTNF mice responded by producing IFN-γ mRNA and chemokines such as CXCL10, CCL5 and CCL7 contributing to T cell migration and granuloma formation (Figure 4B up). However, they succumbed to *M. tuberculosis* infection around 170 days post-infection contrary to WT, which survive to 300 days, confirming that tmTNF is sufficient to control acute, but not chronic infection.

Using the same tmTNF KI mice in a mouse model of *M. tuberculosis* reactivation, it has been reported that tmTNF is not sufficient to support an efficient immunity, although confers protection against acute *M. tuberculosis* infection. However, the long-term protection requires solTNF in order to decrease inflammatory response and to contain *M. tuberculosis* reactivation [123,124]. Moreover, the expression of tmTNF only in T cells (tmTNF-T cells) was shown to be sufficient to confer protection against *M. tuberculosis* infection, but was not associated with a reduction in bacterial load [123] (Figure 4B below).

Several questions about tmTNF signalling are still open because experimental data can be influenced by diverse factors such as the nature of mutations generated on the tmTNF molecule and its impact in the interaction with TNFRs as well as regulatory mechanisms involving TNFRs and their soluble forms.

The impact of the different mutations on the tmTNF molecule has been analyzed at the level of host defense mechanisms against mycobacteria. Indeed, two mouse models of tmTNF KI mice were compared by infecting with a high dose of BCG. tmTNF KI mice with the deletion tmTNFΔ1–9, K11E were able to establish an immune response similar to WT, in contrast, mice with the deletion tmTNFΔ1–12 were highly sensitive to the infection [69]. The authors showed that the difference between the two tmTNF KI mice are at the level of Th1 type immune responses and iNOS activation that tmTNFΔ1–9, K11E develop but not tmTNFΔ1–12 KI mice. Furthermore, the frequency of CD11b+ cells expressing TNFR2 were lower in highly sensitive mice, these authors proposed that that interaction of tmTNFΔ1–12 and TNFR2 could be deficient in contrast to resistant mice [69]. This study also clarified that cellular immune activation can be attributed to reverse signalling of tmTNF characterized by NF-κB activation, NO production and the delivery of IL-6 and RANTES, suggesting that precise regulation of tmTNF activity can be orchestrated by binding with tmTNFR2 or solTNFR2, and an unbalance of this regulatory and complex system can modify the outcome of the disease [69] (Figure 4C). Using a model of pleural tuberculosis, this group has reported that tmTNF but not solTNF regulates the expression of tmTNFR2 on myeloid cells [74].

An interesting report by Keeton and colleagues have shown that soluble or transmembrane forms of TNFRs mediate different activities of TNF. Indeed, solTNFR2 reduces bioactive TNF concentrations through downmodulation of dendritic cell activation, affecting several TNF-dependent functions as cytokines synthesis, whereas tmTNFR2 promotes immune protection [125].

Myeloid-derived suppressor cells (MDSC) have been described as natural suppressor cells inhibiting T-helper lymphocytes’ proliferative response. A study has reported a new mechanism to explain the role played by tmTNF in the control of BCG infection. BCG-infection induces MDSC accumulation, and tmTNF expression on MDSC is crucial to activate their suppressive function. Chavez-Galan et al. [126] suggested that tmTNF on MDSC interacts specifically with TNFR2 on CD4 T cells (Figure 4D). The suppressive activity of MDSC attenuates the excessive inflammation associated with mycobacterial infection [125]. A limitation of this study is that the intracellular pathways are not yet clarified. Using a tumor model, it has been reported, that tmTNF on MDSC activated MDSC upregulating arginase-1 and iNOS transcription to promote NO, IL-10, and TGF-β secretion and enhancing the inhibition of lymphocyte proliferation [127]. It is possible that MDSC in a BCG-infection model also can upregulate the same molecules, but the exact pathway is still unclear.

## 6. TNF Apoptosis Inhibition in Macrophage-Mycobacterial Infection

The apoptotic process in *M. tuberculosis* infected macrophages could be initiated by TNF-TNFR1 interaction with TRADD (TNFR-associated death domain) and FADD (Fas-associated death domain) association and subsequent aggregation of death effector domains (DEDs), procaspase-8 activation and the DISC (death-inducing signalling complex) assembly. Finally, effector caspases are activated. TNF-induced apoptosis through molecules receptor-associated that impact caspase activation, resistance to cell death may be influenced by NF-kB [128].

Virulence of mycobacteria is strongly associated with cell death evasion during infection. Virulent strains like *M. tuberculosis* induce a miR-30A overexpression in infected macrophages, inhibiting the autophagy and negatively impacting the immune control [129]. A particular inverse correlation has been found between mycobacteria virulence and apoptosis induction [130]. In this regard, recently it has been reported that TNFR2 increases microbicidal activity against *M. tuberculosis* independently of IFNγ and nitric oxide, and it displays an inverse correlation with macrophages apoptosis, but this apoptosis is not observed under BCG infection, suggesting that regulation of apoptosis and mycobacterial replication by TNFR2 is a virulence dependent pathway [131]. However, beyond virulence, several factors are involved with the induction or inhibition of apoptosis during mycobacterial infection, for instance, strain phenotype, stage of infection, and cell condition may interfere. Other mechanisms have been observed, such as soluble TNFR2 secretion for blocking TNF activities including apoptosis and reducing Fas receptors expression affecting Fas-ligand cell death [132,133].

In an infection model with *M. tuberculosis* H37Rv in U937 and THP-1 cell lines and in human monocyte-derived macrophages, apoptosis inhibition involves the extrinsic apoptosis pathway, but it does not interfere with the mitochondrial apoptosis pathway [134]. Two proteins, Rv3654c and Rv3655, secreted by *M. tuberculosis* into the cytoplasm were identified, as responsible for apoptosis suppression. Rv3654c cleaves PFS (polypyrimidine tract binding Protein-associated Splicing Factor), which decreases the expression of caspase-8 [134]. In another way, Rv3655c is associated with high expression of ALO17, a protein commonly associated with ALK (anaplastic lymphoma kinase). Associated proteins have been involved in anti-apoptotic events, in particular, in the inhibition of PI3/Akt and caspases [135]. However, the role that the intrinsic pathway may play during *M. tuberculosis* infection is not clear. It has been suggested that mitochondrial apoptosis pathway may be a strategy to induce necrosis in infected macrophages during a well-defined stage of the infection [134,136].

A different mechanism has been proposed by Miller et al. NuoG a subunit of NDH-1 (type I NADH dehydrogenase) is involved in neutralization of NOX2-derived reactive oxygen species (ROS), promoting inhibition of TNF-induced apoptosis [137]. If ROS is increased in the phagosome, apoptosis in the macrophage can be activated. Indeed, several links between apoptosis and TNF activated pathway have been described affecting caspase-8 activation using kinases ASK1m p38 and c-Abl, promoting FLIPS degradation by the proteasome and finally activating procaspase-8 [138]. Mycobacterial apoptogenic moieties have been reported, particularly PstS-1, a 38-kDa lipoprotein of *M. smegmatis,* involving TNF and FasL activation, which upregulates TNFR1, TNFR2 and Fas. In this process, TLR2 was involved as well as in the activation of caspase-8, caspase-9 and caspase-3 [139]. In summary, a variety of apoptotic ways of activation may depend on TNF; however, particular conditions of mycobacterial infection may contribute to inhibition or efficiently programmed cell death of the infected cell.

## 7. Conclusions

TNF is a key regulatory cytokine that plays an important role in the innate and adaptive immune responses during mycobacterial infections. However, several mechanisms can influence the course of the infection. The first interactions of TNF and TNF receptors after mycobacterial phagocytosis may play a crucial role inside a specific environment. It is also of interest to consider the balance between the two TNF forms, tmTNF, and solTNF and cells responding to these TNF forms that can influence the outcome of mycobacterial infections.

Recent evidence has pointed out the relevance of tmTNF-mediated reverse signalling during mycobacterial infections. It is of interest that this pathway not only favors proinflammatory responses, as discussed in this review, but tmTNF attenuates Th1 cell-mediated inflammatory responses and mediates cell recruitment. These are new insights elucidating tmTNF/TNFRs axis that can be considered as target to control mycobacterial infections, mainly focusing on TB, which still as a major public health worldwide.

Moreover, the selective inhibition of solTNF is another important target because it improves survival in an acute infection, nevertheless diverse signalling mechanisms for both forms of TNF, TNFR1 and TNFR2 receptors remain to be elucidated. More research on the biology of TNF is required to design improved therapies that alleviate inflammatory diseases while maintaining protection against mycobacteria. Furthermore, this will lead to personalized treatment as a promise to improve the clinical outcome of patients suffering from mycobacterial infections.

## Figures and Tables

**Table 1:** Selective TNFR1 and TNFR2 inhibitors under development.

Target	Inhibitor	Class	Reference
TNFR1	XPro1595	TNF mutein	[93]
	Atrosab	Humanized IgG1	[101]
	R1antTNF R1antTNF-T8 scR1antTNF	TNF muteins	[102,103,104]
	DMS5540	Bispecific fusion protein	[105]
	AlbudAb	Antibody	[106]
	TROS (TNF Receptor- One Silencer)	Nanobody (Nb) technology	[107]
	PMG (physcion-8-O-β-D-monoglucoside)	Small molecule	[108]
	PLAD (pre-ligand assembly domain)	Small molecule	[109]
	ASO	Antisense oligonucleotides	[109]
	GSK1995057	Antibody	[110]
	Atrosimab	Atrosab monovalent form	[111]
	Zafirlukast, DS42	Small molecule	[112]
TNFR2	Antagonist Ab	Antibodies	[113,114]

## Data Availability

Not applicable.

## Abbreviations

ACE2	Angiotensin-Converting Enzyme-2
ADAM17	A Disintegrin and Metalloproteinase (ADAM) domain 17
AICD	Activation-Induced Cell Death
ASK1	Apoptosis Signal-Regulating Kinase 1
CAV1	Caveolin-1
DD	Death Domain
DEDs	Death Effector Domains
DISC	Death-Inducing Signalling Complex
ER	Endoplasmic Reticulum
FADD	Fas-Associated Death Domain
FasL	Fas ligand or CD95L
FcγR	Fc region of IgG
HIF-1α)	Hypoxic-Inducible Factor-1 subunit α
IKK	Ikappa B kinase
IKK-α	Subunit α del IκB kinase complex (also IKK1)
IKK-β	Subunit β del IκB kinase complex (also IKK2)
IKK-γ	Subunit γ (also NEMO)
iNOS	Inducible Nitric Oxide Synthase
iRHOM2	Rhomboid 5 homolog 2
JNK	c-Jun-N-terminal kinase
LS	Leader Sequence
MAP3K	Mitogen-Activated Protein Kinase Kinase Kinase
MAPK	Mitogen-Activated Protein Kinases
MAPKAP kinase-2	MAP-kinase-activated protein kinase-2
MDSC	Myeloid-Derived Suppressor Cells
NDH-1	Type I NADH Dehydrogenase
NF-κB	Nuclear Factor kappa B
NRD	Nardilysin
PGRN	Progranulin
PI3K	Phosphatidylinositol 3-kinase
PKC	Protein kinase C
RANTES	Regulated on Activation Normal T Cell Expressed and Secreted chemokine
RIPK1	Receptor Interacting Protein Kinase 1
RIPK1^U^	Ubiquitination of RIPK1
ROS	reactive oxygen species
solTNF	Soluble TNF
Src	Sarcoma kinase
TACE	TNF Converting Enzyme
TGF-β	Transforming growth factor-beta
tmTNF	Cell membrane anchored TNF or transmembrane TNF
TNF	Tumor Necrosis Factor
TNFR1	TNF receptor 1
TNFR2	TNF receptor 2
TRADD	TNFR1-Associated Death Domain
TRAF2	TNFR-Associated Factor 2
Uev1A	Ubiquitin (Ub)-conjugating enzyme variant 1A
ZBG	Zinc Binding Groups
